# Regulatory role of KCa3.1 in immune cell function and its emerging association with rheumatoid arthritis

**DOI:** 10.3389/fimmu.2022.997621

**Published:** 2022-10-05

**Authors:** Yi Lin, Ying-Jie Zhao, Hai-Lin Zhang, Wen-Juan Hao, Ren-Di Zhu, Yan Wang, Wei Hu, Ren-Peng Zhou

**Affiliations:** ^1^ Department of Clinical Pharmacology, The Second Hospital of Anhui Medical University, Hefei, China; ^2^ Inflammation and Immune Mediated Diseases Laboratory of Anhui Province, Anhui Institute of Innovative Drugs, School of Pharmacy, Anhui Medical University, Hefei, China; ^3^ The Key Laboratory of Anti-inflammatory and Immune Medicine, Ministry of Education, Anhui Medical University, Hefei, China

**Keywords:** KCa3.1, immune cells, joint inflammation, rheumatoid arthritis, synovitis

## Abstract

Rheumatoid arthritis (RA) is a common autoimmune disease characterized by chronic inflammation. Immune dysfunction is an essential mechanism in the pathogenesis of RA and directly linked to synovial inflammation and cartilage/bone destruction. Intermediate conductance Ca^2+^-activated K^+^ channel (KCa3.1) is considered a significant regulator of proliferation, differentiation, and migration of immune cells by mediating Ca^2+^ signal transduction. Earlier studies have demonstrated abnormal activation of KCa3.1 in the peripheral blood and articular synovium of RA patients. Moreover, knockout of KCa3.1 reduced the severity of synovial inflammation and cartilage damage to a significant extent in a mouse collagen antibody-induced arthritis (CAIA) model. Accumulating evidence implicates KCa3.1 as a potential therapeutic target for RA. Here, we provide an overview of the KCa3.1 channel and its pharmacological properties, discuss the significance of KCa3.1 in immune cells and feasibility as a drug target for modulating the immune balance, and highlight its emerging role in pathological progression of RA.

## Introduction

Rheumatoid arthritis (RA) is an autoimmune disease that primarily affects the joints. The average global incidence is 0.5% to 1.0%, with genetic factors accounting for approximately 60% risk of RA (1). The primary goal of RA therapy is to restore the immune balance and reduce synovial inflammation and joint damage. The traditional drug of RA ranges from disease-modifying anti-rheumatic drugs (DMARDs) (eg, methotrexate and Janus kinase inhibitor tofacitinib) to biologic agents (eg, tumor necrosis factor inhibitors) and some adjuvant therapy drugs like non-steroidal anti-inflammatory drugs (NSAIDs) and glucocorticoids (GC) (1). However, the currently available drugs provide limited long-term efficacy along with increased risk of severe side-effects. Therefore, management of RA remains a topic of considerable research focus. In this context, we propose potential novel strategies for the treatment of RA by searching for targets that restore the balance of immune function.

KCa3.1, a Ca^2+^-activated intermediate conductance K^+^ channel regulated by the Ca^2+^-binding protein calmodulin (CaM), was first identified in erythrocytes by Gardos in 1958 (therefore also designated the Gardos channel) (2). The channel is encoded by the *KCNN4* gene and directly pre-associated with CaM in the absence of Ca^2+^. When the intracellular free Ca^2+^ concentration is higher than 100 nM, the KCa3.1 channel is activated after Ca^2+^ binds to CaM (3). This results in increased K^+^ efflux and change in membrane potential, providing a driving force for Ca^2+^ influx. Physiological and pharmacological studies have shown that the KCa3.1 channel modulates membrane potential and Ca^2+^ signaling in activated T and B cells, macrophages and fibroblasts (4). From a pathological perspective, the KCa3.1 channel is abnormally opened to maintain Ca^2+^ homeostasis, thereby regulating various cellular functions ranging from proliferation and differentiation to migration (5). Therefore, the KCa3.1 channel may serve as a potential therapeutic target for diseases associated with cell activation and hyperproliferation, such as diabetic nephropathy (6), ulcerative colitis (7), and RA (8).

The pathological process of RA involves interactions of multiple immune cells, synovial fibroblasts, cytokines, and proteases. Synovial tissue gradually develops chronic inflammation that progresses to cartilage damage and bone erosion, leading to joint damage and multiple clinical symptoms (1, 9). Several studies have provided evidence that KCa3.1 contributes substantially to immune imbalance in RA. Notably, obstruction of the KCa3.1 channel effectively inhibits disease progression by alleviating immune inflammation and joint damage, suggestive of its significant therapeutic value in RA. This article provides a summary of the current information on the immunoregulatory mechanisms related to KCa3.1, its functional roles in the development of RA, and potential as a pharmacological target for disease management.

## Overview of KCa3.1

**Figure 1:**
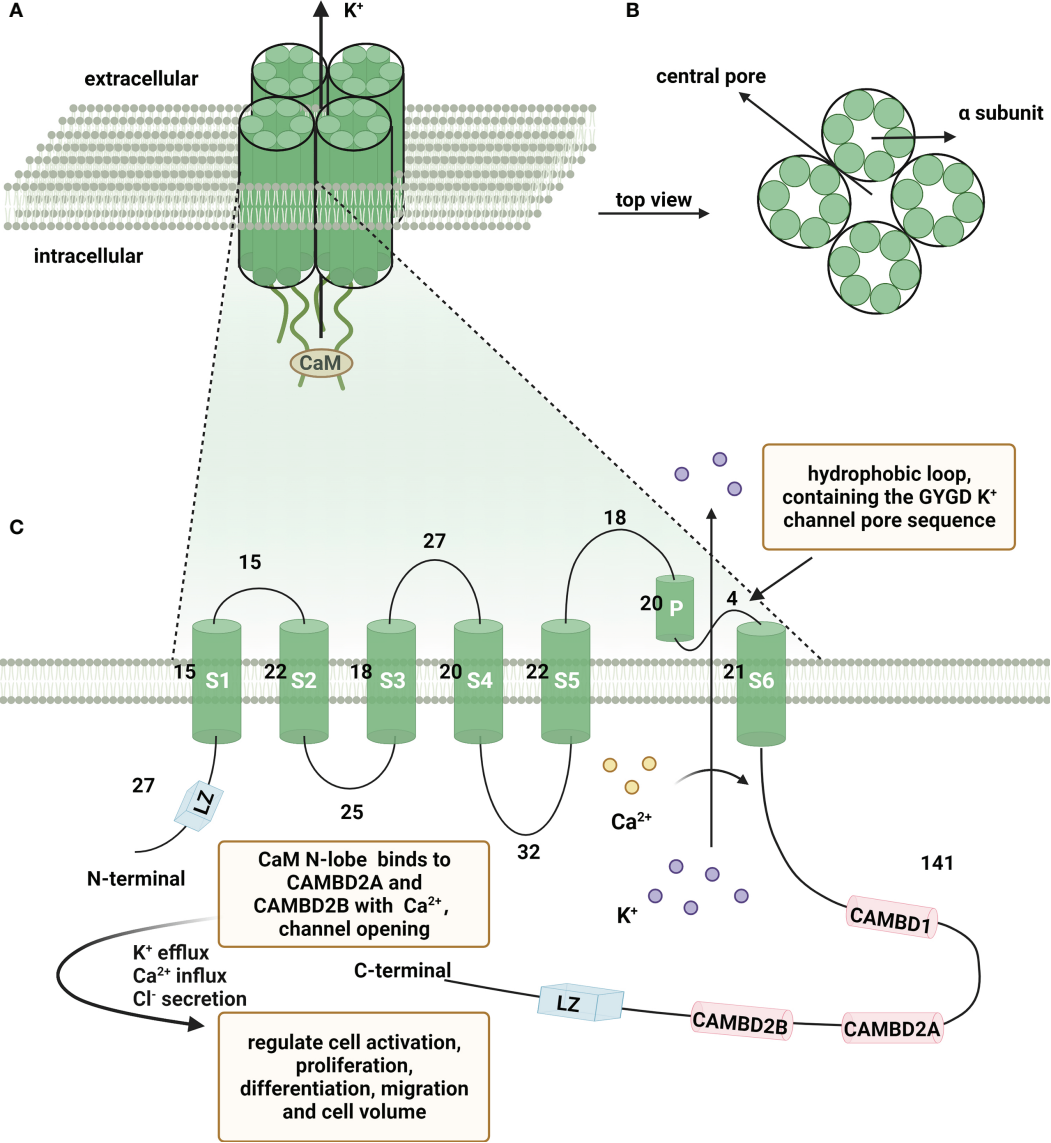
Schematic representation of the structure of KCa3.1. A functional Ca^2+^-activated intermediate conductance K^+^ channel (KCa3.1) comprises four α subunits organized around a central pore through which K^+^ flows out of the cell. **(A)** KCa3.1 channel composed of four α subunits. **(B)** Top view of four α subunits around the central pore. **(C)** Schematic representation of a single KCa3.1 subunit, showing a total of 427 amino acids and consists of six transmembrane segments, named S1-S6. The K^+^ ion conduction pore is located between the loop and S6, containing the GYGD K^+^ channel pore sequence. CaM N-lobe binds to CAMBD2A and CAMBD2B with Ca^2+^, leading to channel opening. (Created with BioRender.com).

KCa3.1 is a multifunctional intermediate conduction channel also known as IKCa1, SK4, IK-1 or KCa4 (10, 11). This channel belongs to a gene family consisting of all Ca^2+^-activated K^+^ channels. The International Union of Pharmacology has now classified the gene family into three groups: KCa1.1 (BK, big-conductance K^+^ channel), KCa2.1, KCa2.2, KCa2.3 (SK, small-conductance K^+^ channel) and KCa3.1 (IK, intermediate-conductance K^+^ channel) (12). KCa3.1 is a membrane-spanning protein composed of four α-subunits (13). Each α-subunit has six transmembrane segments (S1-S6) with a pore motif between S5 and S6. The pore region is formed by the transmembrane helices S5 and S6 in the symmetrical center of the tetramer, generating a K^+^ conduction pathway (14). In genetics, the coding gene *KCNN4* is located at the q13.2 locus of human chromosome 19 (15, 16). The encoded protein contains 427 amino acids with a short N-terminal domain and long C-terminal tail. The C-lobe of CaM constitutively binds to CAM-binding domain (CAMBD) 1 (positions 312-329) in a Ca^2+^-independent manner at the C-terminus of KCa3.1, whereas the CaM N-lobe barely binds to the channel and its binding pocket remains closed. In the presence of high Ca^2+^, the N-lobe of CaM binds with Ca^2+^ and rearranges into an open conformation. The N-lobe of CaM pulls the S45A (first helix of the S4-S5 linker) helix down, keeping the S45B (tightly coupled to the pore-lining S6 helix) away from the pore axis. This expands the S6 helical bundle and eventually opens the pore (17). The N-lobe of CaM binds to KCa3.1 at CAMBD2A (a nearby segment, positions 344-353) in the same subunit and CAMBD2B (a distal segment, positions 360-373) in an adjacent subunit (18). Furthermore, a pivotal role of channel tetramerization and trafficking of two leucine zipper (LZ) motifs in the N- and C-termini has been reported (19, 20). The structure of KCa3.1 is shown schematically in **Figure 1**.

Here we focus on the transcriptional regulation, spliceosome regulation and epigenetic regulation of KCa3.1 (21). At the transcriptional level, activation protein-1 (AP-1) in conjunction with transcription factor Ikaros-2, was demonstrated to enhance KCa3.1 channel expression, which promoted the mitogenesis of preactivated lymphocytes (22). Additionally, laminar shear stress upregulates endothelial KCa3.1 by binding of AP-1 and cAMP response element (CRE) to promoter in a CaMK/Akt/p300 pathway-dependent manner (23). Mutation of the AP-1 binding motif in T cells as well as the transfection of AP-1 decoy oligonucleotides into cardiac fibroblasts were shown to significantly downregulate the expression of KCa3.1 (22, 24). Furthermore, two NF-κB binding sites were identified in the promoter region of KCa3.1, and the up-regulation of KCa3.1 in colon cancer cells was mediated in an NF-κB-dependent manner (25). A functional repressor element 1-silencing transcription factor (REST or NRSF) was confirmed to be a negative regulator of KCa3.1 transcription (26). In a study on tumors, histone deacetylase 2 (HDAC2) and HDAC3 were found to downregulate KCa3.1 transcript levels in a REST-independent and insulin-like growth factor-binding protein 5 (IGFBP5)-independent manner in the breast cancer cell line, TMB-1 (27). Meanwhile, HDAC2 and HDAC3 were found to be involved in the epigenetic regulation of KCa3.1 in the KCa3.1-expressing human prostate cancer cell line, PC-3. Epigenetically, *KCNN4* is hypermethylated in memory B cells in common variable immunodeficiency (CVID) individuals relative to healthy individuals (28). However, in a genome-wide DNA methylation analysis, Bulk et al. found that the *KCNN4* promoter was hypomethylated in lung cancer (29). Besides, Ohya et al. identified novel spliced variants of KCa3.1 (human(h) KCa3.1b) from the human thymus, which differs from hKCa3.1a for the lack of the N-terminal domains. The study suggests that the N-terminal domain of KCa3.1 is essential for channel trafficking to the plasma membrane (30). Moreover, Du et al. showed that *KCNN4* was regulated by several microRNAs, such as miR-204-5p studied in the research of pancreatic ductal adenocarcinoma (PDAC) (31).

KCa3.1 is located in the lung, distal colon, and immune-related tissues, such as thymus, bone marrow, and lymph nodes (32). In-depth studies have shown that KCa3.1 is almost expressed in non-excitable cells, such as fibroblasts, lymphocytes, and other immune cells. At the cellular level, electrophysiological and pharmacological characterization studies have identified the presence of KCa3.1 in plasmalemma and mitochondrial membrane (33). KCa3.1 channels are additionally voltage-independent and unaffected by membrane potential, with Ca^2+^-dependent and inwardly rectifying properties of intermediate conduction (34). Functionally, basolateral KCa3.1 provides the driving force for Cl^-^ secretion induced by activators such as Ca^2+^ in human and rat colon (35). KCa3.1 is also involved in regulation of cell volume in lymphocytes (36). Similarly, patch-clamp studies showed that the CFT1-LCFSN cell, a cystic fibrosis airway cell line, copes with hypotonic challenge *via* increasing the KCa3.1 current (37). Moreover, the KCa3.1 channel is activated at elevated cytosolic Ca^2+^ concentrations of above 100 nM. Substantial activation of the KCa3.1 channel leads to K^+^ efflux, thereby restoring and stabilizing the fully hyperpolarized membrane potential to maintain a continuous driving force of Ca^2+^ influx (38). While Ca^2+^ is indispensable for various physiological activities of the body, continuous influx is necessary for activation, proliferation and other physiological function of immune cells and cytokine production (39). Thus, the functions of KCa3.1 described above suggest the potential of targeting KCa3.1 in the treatment of diseases associated with immune imbalance.

## Activators and inhibitors of KCa3.1

The pharmacological effects of the KCa3.1 channel have been widely explored and its activators and inhibitors analyzed in various diseases (**Table 1**). The majority examples of channel activators are documented in the literature related to cardiovascular diseases, neurological diseases and immune diseases. For instance, 1-ethyl-2-benzimidazolidinone (1-EBIO) serves as a direct and potent specific activator of KCa3.1 *via* increasing sensitivity of the channel to resting levels of Ca^2+^ (43). A dichloro analog of benzimidazolidinone, 5,6-dichloro-1-EBIO (DC-EBIO), is reported to be 30 times more potent than EBIO (44). Naphtho [1, 2-d] thiazole-2-ylamine (SKA-31) and its optimized product, 5-methylnaphtho [2,1-d] oxazole-2-amine (SKA-121), act in a similar manner to EBIO (45). Another preliminary study showed for the first time that 6,7-dichloro-1H-indole-2,3-dione-3-oxime (NS309) positively regulates KCa3.1 with higher potency and selectivity than 1-EBIO in the HEK-293 cells (human embryonic kidney cells). The above findings indicate that NS309 presents an excellent alternative to 1-EBIO as a pharmacological tool in KCa3.1 activation-related research (46). In addition, chlorzoxazone (CZ) and zoxazolamide (ZOX) are often used clinically as pharmacological activators of the KCa3.1 channel and have entered Phase IV and Phase II clinical trials, respectively (51). Classical methylxanthine compounds, including theophylline, 3-isobutyl-1-methylxanthine (IBMX) and caffeine, are reported to interact directly with channel proteins to activate KCa3.1 (47). Gerlach et al. demonstrated that ATP activates KCa3.1 in excised, inside-out patches in a protein kinase A inhibitor 5-24-dependent manner (52). In their experiments, ATP specifically activated chimera containing the KCa3.1 C-terminal amino acids His^299^-Lys^427^, but not other highly homologous Ca^2+^-activated K^+^ channels. In terms of indirect activation, the human single cAMP-dependent protein kinase (PKA) site (S334A) on the KCa3.1 α subunit is dependent on phosphorylation of PKA to reduce binding of CaM to the KCa3.1 channel. PKA signaling pathway inhibitors, such as PKI14-22, Rp-8-Br-cAMPS, and N-[2-(4-bromocinnamylamino) ethyl]-5-isoquinoline (H-89), significantly reversed downregulation of KCa3.1 channel, thereby restoring its function, while Sp-8-Br-cAMPS, a PKA activator, exerted the opposite effect (48, 53). Interestingly, PKA-mediated phosphorylation was shown to have no regulatory effect on KCa3.1 channel in the above study (50). Moreover, a monoclonal blocking antibody against programmed death 1, pembrolizumab, has been identified that promotes KCa3.1 activity and concomitantly increases Ca^2+^ flux in cytotoxic T cells of patients immediately after treatment (54, 55). In conclusion, most KCa3.1 channel activators have low potency and poor selectivity and modulate other ion channels simultaneously.

**Table 1:** Inhibitors and Activators of KCa3.1.

Activators/Inhibitors	Substances	Structure/formula	IC_50_/EC_50_	Experimental cells	Description	Clinical trial	References
**Channel activators**	1-EBIO	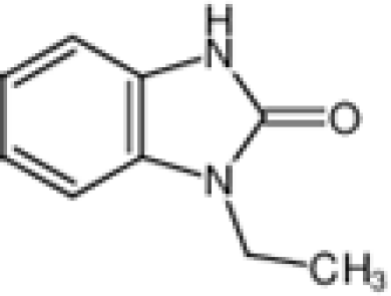	84 μM(EC_50_)	Xenopus oocytes	Increase channel open rate	\	(29, 32)
	DC-EBIO	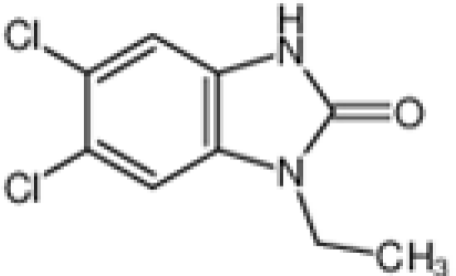	Test at 100 μM	Capan-1 cells	Increase channel open rate	\	(40, 41)
	SKA-31	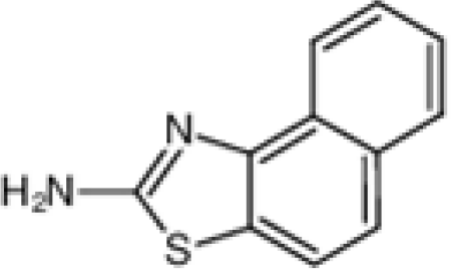	260 nM (EC_50_)	COS-7 cells	open channel	\	(30)
	SKA-121	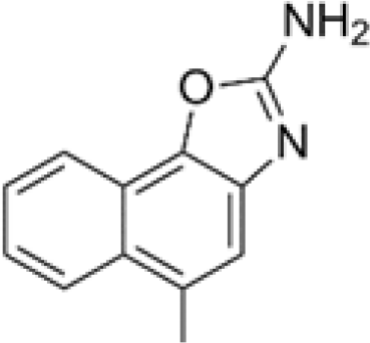	110 nM (EC_50_)	COS-7 cells	open channel	\	(30)
	NS309	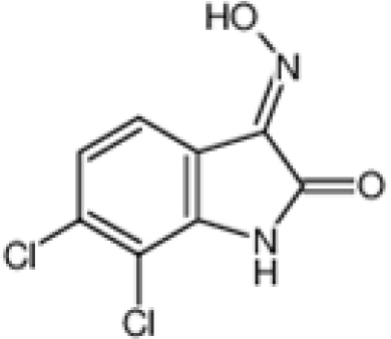	10 nM (EC_50_)	HEK-293 cell	open channel	\	(31)
	CZ	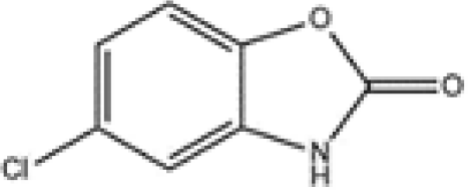	98 μM(EC_50_)	Xenopus oocytes	Increase channel open rate	Phase I-IV	(32)
	ZOX	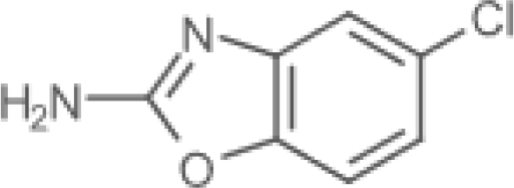	Test at 300 μM	Xenopus oocytes	Increase channel open rate	Phase I-II	(32)
	theophylline	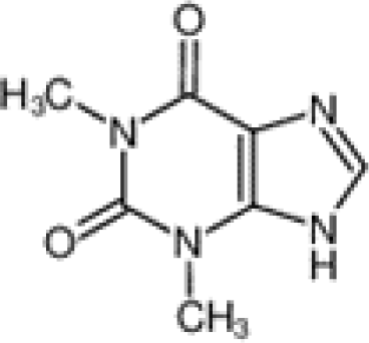	Test at 0~1500 μM	HEK-293 cell	Mandatory Ca^2+^-dependent , independent of phosphorylation	Phase I-IV;	(33)
**Channel activators**	IBMX	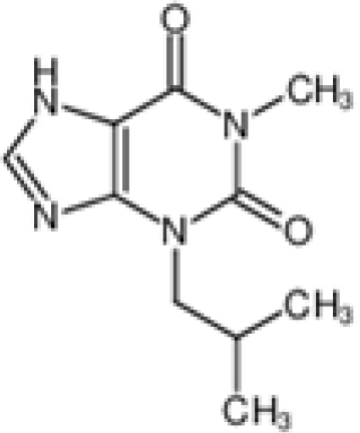	Only test at 1mM	HEK-293 cell	Mandatory calcium dependence	\	(33)
	Caffeine	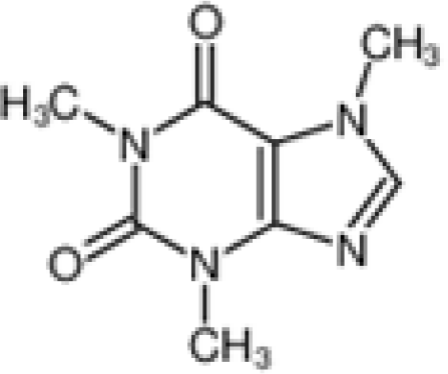	Only test at 1mM	HEK-293 cell	Mandatory calcium dependence	Phase I-IV	(33)
	ATP	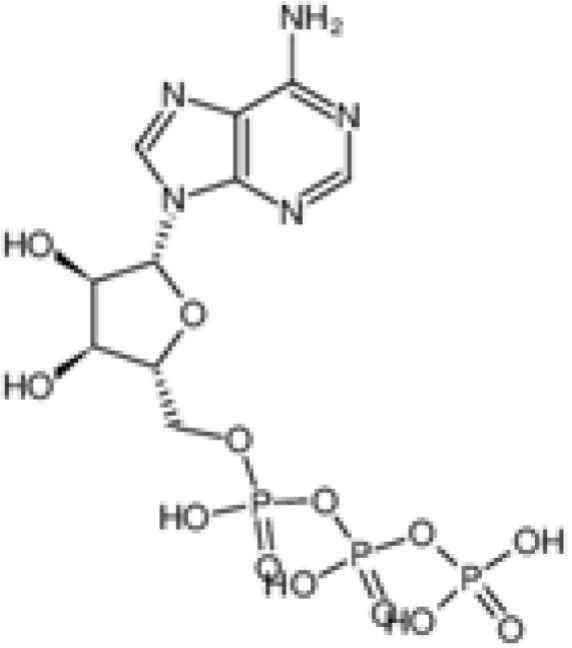	Only test at 100 μM	Human microglia	activate purinergic receptors, free [Ca^2+^]i ↑	Phase I-IV	(42)
	PKI14-22	C_53_H_100_N_20_O_12_	Only test at 10M	MLS-9 microglia, primary rat microglia	Inhibit PKA, increase current	\	(35)
	Rp-8-Br-cAMPS	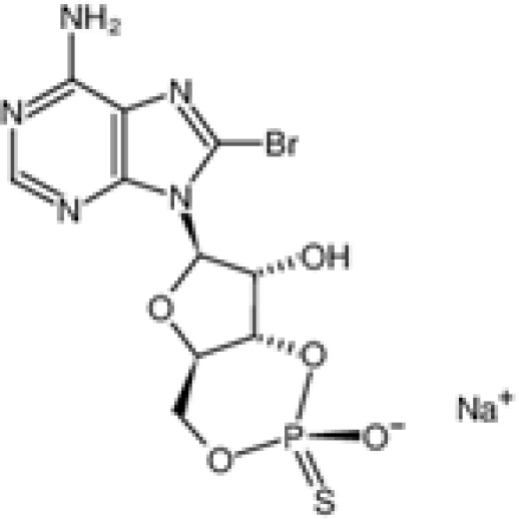	Test at 10M, 100μM	MLS-9 microglia, HEK-293 cell, post-SE neurons	Inhibit PKA, increase current	\	(35, 36)
	H-89	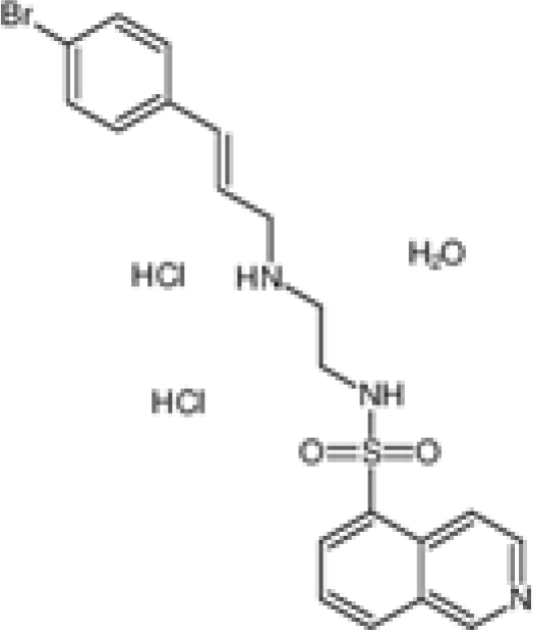	Test at 1μM, 10 μM	Post-SE neurons, HEK-293 cell	Inhibit PKA	\	(36)
**Peptide inhibitors**	ChTX-Glu^32^	C_175_H_272_N_56_O_57_S_7_	250 nM (E_max_)	Human T-lymphocytes	Salt bridge anchors the outer vestibule	\	(43, 44)
	MTX	C_145_H_231_N_45_O_47_S_8_	1.4 nM (IC_50_)	CHO cells	Selective inhibitor	\	(45)
	α-KTx6	\	\	\	Inhibit KCa3.1 with nanomolar affinity	\	(46)
**Small molecule inhibitors**	clotrimazole	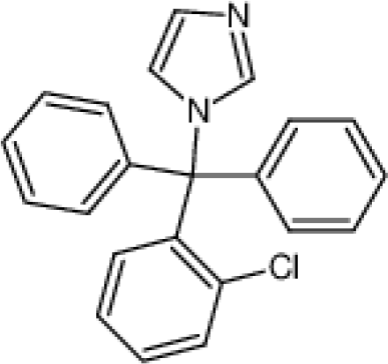	3±0.5 μM (EC_50_)	T cell	inhibit mitosis	Phase I-IV	(47)
	TRAM-34	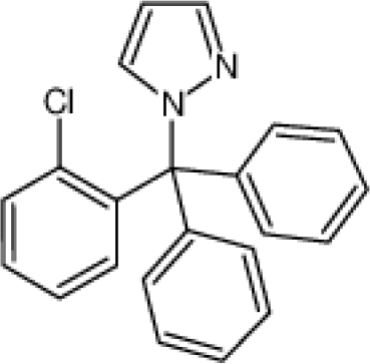	5.5±0.5 μM(EC_50_)	T cell	inhibit mitosis	\	(47)
	NS6180	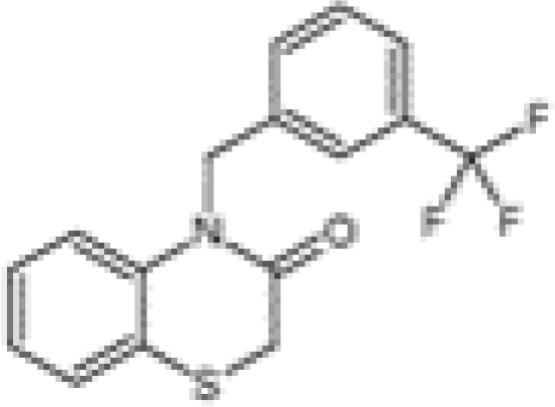	Human: 14 Nm; Mouse: 15 nM; Rat: 9 nM (IC_50_)	human, mice, and rat erythrocytes	binding amino acid	\	(48, 49)
	ICA-17043	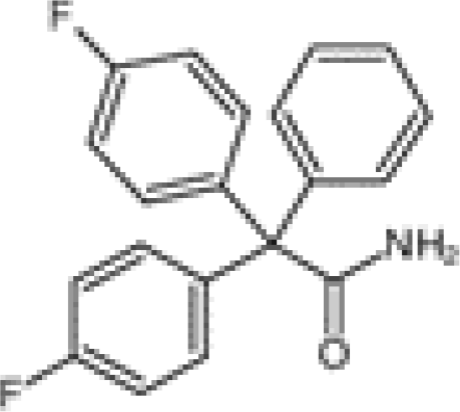	11±2 nM (IC_50_)	Human erythrocytes	High selective	Phase I-III	(50)
	4-Phenyl-4H-pyran	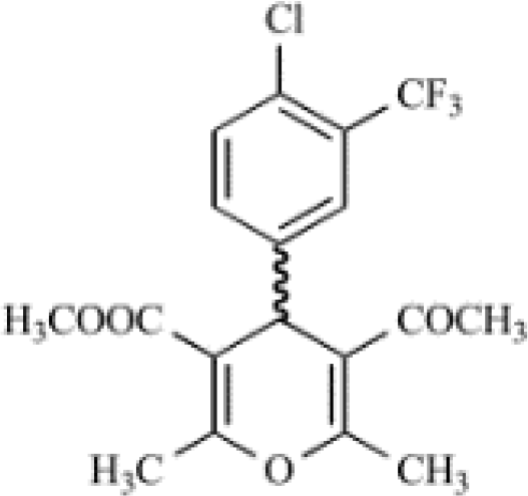	8nM (IC_50_)	C6BU1 rat glioma cells	Inhibit ion conduction directly	\	(20, 48)

KCa3.1 channel inhibitors are divided into two main categories: peptides and small molecule inhibitors. Peptide blockers bind to the outer vestibule of the channel and form multi-point contacts with channel residues whereas small-molecule blockers pass through the membrane and bind the cavity from the inside, blocking K^+^ outflow (38). The majority of KCa3.1 channel peptide inhibitors are toxin polypeptides. The most common is the scorpion toxin Glu32-charybdotoxin, initially isolated from *Leiurus quinquestriatus*. Nevertheless, the scorpion toxin peptide has low selectivity for KCa3.1 and additionally shows activity against both KCa1.1 and Kv1.3 channel (a voltage-gated K^+^ channel) (56, 57). Maurotoxin (MTX) (58) and urotoxin (α-KTx6) (59) display affinity for KCa3.1 but also affect the Kv1.2 channel (a voltage-gated K^+^ channel). Accordingly, toxin polypeptide KCa3.1 channel blockers have limited experimental value for *in vivo* research on KCa3.1 due to their low specificity and are more commonly used to investigate the pharmacological properties of KCa3.1 *in vitro (* 60).

Small-molecule inhibitors of KCa3.1, primarily derived from the antibacterial drug clotrimazole, effectively block the channel and inhibit mitosis of activated prolymphocytes (22). However, clotrimazole inhibits cytochrome P450 enzymes *in vivo*, causing severe side-effects, which limits its pharmaceutical value (45). A derivative inhibitor of clotrimazole, 1-[(2-chlorophenyl) diphenylmethyl]-1H-pyrazole (TRAM-34), was further developed, which could avoid the adverse reactions of cytochrome P450 enzyme inhibition (61). TRAM-34 is the most commonly used KCa3.1 channel inhibitor in pharmacological experiments. Mechanistically, TRAM-34 binds threonine 250 and valine 275 in the pore cavity of the KCa3.1 channel, preventing penetration of ion (62).4-[[3-(trifluoromethyl) phenyl] methyl]-2H-1, 4-benzothiazin-3(4H)-one (NS6180) inhibits KCa3.1 channel activity using the same mechanism as TRAM-34 but has low bioavailability and is therefore only suitable for topical therapy. Senicapoc, also known as ICA-17043, is a potent and selective blocker of KCa3.1. Compared to other receptors, senicapoc displays higher selectivity for KCa3.1 and lower possibility of off-target effects (63, 64). A number of novel compounds have been synthesized using the L-type Ca^2+^ channel blocker nifedipine as the template, such as cyclohexadiene 4 (32) and the nano-affinity KCa3.1 channel inhibitor cyclohexadiene lactone composed of cyclohexadiene (4), and phenyl-4H-pyran. Due to the difficulty in synthesizing phenyl-4H-pyran and its short half-life after intravenous injection (62), the compound is not a suitable replacement for TRAM-34 as a KCa3.1 inhibitor. Clinically, the antihypertensive drug nitrendipine blocks KCa3.1 channel at a dose of 100 nM (65). Previous studies have shown that in the PI3K-PI(3)P signaling pathway, LY29400259 (a phosphatidylinositol 3-kinase inhibitor) (55) and ellagic acid (a nucleoside diphosphate kinase B kinase inhibitor) (66), prevent phosphorylation of specific group amino acid and inhibit activity of the KCa3.1 channel. Recently, Licochalconer A, a chalcone compound extracted from licorice, was shown to block KCa3.1 in a concentration-dependent manner, with anti-inflammatory effects (67). In general, the pharmacological effects of the KCa3.1 channel are relatively well characterized and meet the pharmacological needs in the relevant studies. However, the most rigorous obstacle to clinical application of KCa3.1 modulators is almost associated with their low selectivity, so it is of great significance to explore highly specific drugs targeting KCa3.1 for conforming to clinical use.

## Abnormal expression of KCa3.1 in rheumatoid arthritis

RA is an autoimmune disease characterized by inflammation of the synovium, with the essential site of inflammation identified as the synovial lining. In the process of lymphocyte activation and pathological function in rheumatoid arthritis, the increase of transient intracellular free calcium level plays a crucial role. A study have found that compared with healthy people, RA patients have elevated basal cytoplasmic free calcium level ([Ca^2+^]_cyt_) and abnormal activation of KCa3.1 channel to maintain calcium influx in peripheral T lymphocytes (68). Additionally, Ca^2+^-activated K^+^ currents with the characteristics of KCa3.1 channel were detected in synovial fibroblasts from RA patients. TGF-β1-induced KCa3.1 overexpression stimulates the proliferation and mediator secretion of synovial fibroblasts, which can be suppressed by KCa3.1 inhibitors. This result supports the theory that KCa3.1 is closely related to synovial inflammation (8). In addition, *KCNN4* is required for fusion of macrophages to form osteoclasts or multinucleated macrophages (MGCs) during the immune response to RA (69). KCa3.1 is expressed in both physiological and inflammatory osteoclast formation and is the only channel in the Ca^2+^-activated K^+^ channel family that is upregulated during the process of receptor activator of nuclear factor-κB ligand (RANKL)-induced osteoclast formation. The collective results confirm an association of abnormal expression of KCa3.1 with pathogenesis of RA.

Experimental studies on animal models suggest that KCa3.1 is significantly associated with inflammation and pathogenesis of RA. In a collagen antibody-induced arthritis (CAIA) model, alleviated joint inflammation and tissue damage was observed in *KCNN4* ^-/-^ mice compared to *KCNN4* ^+/+^ mice (69). One extremely interesting phenomenon was that collagen-induced arthritis (CIA) *KCNN4* ^-/-^ mice did not develop autoimmune arthritis (70). Specifically, following intradermal injection of chicken collagen type II into the base of the tail of *KCNN4* ^-/-^ mice on days 0 and 21, no *KCNN4* ^-/-^ mice developed clinical evidence or histological signs of arthritis, in contrast to wild-type mice. Notably, the CIA *KCNN4* ^-/-^ model indicates a possible pro-inflammatory effect of KCa3.1 in RA. However, the specific mechanisms by which deficiency of *KCNN4* induces resistance against joint inflammation in CIA models remain unclear. These findings suggest that targeting KCa3.1 deficiency may alleviate joint inflammation and limit the development of persistent joint damage in experimental animal models, presenting a potential strategy for RA therapy.

## Regulatory roles of KCa3.1 in immune cells

### Role of KCa3.1 in T cells

The most prominent cell type in immune diseases is the T cell, which is responsible for recognizing antigens and generating immune responses. During pathogenesis of RA, autoantigens are presented to T cells by antigen-presenting cells. Following activation of pathogenic self-reactive T cells, various innate immunocytes are activated. Immediately afterwards, inflammatory signaling pathways are initiated, secreting various cytokines to trigger synovial tissue inflammation. KCa3.1 expressed in T cells initiates expression of genes that promote T cell activation and proliferation (71). Notably, stimulated activated T cells express significantly higher levels of KCa3.1 than resting T cells (22).

CD4^+^ T cells are core cells of the immune system, coordinating the adaptive immune response and regulating immune and non-immune cell functions through cytokine production (72, 73). In CD4^+^ T cells, the KCa3.1 channel is activated mainly through the phosphatidylinositol 3 phosphate (PI(3)P) signaling pathway. After antigen presentation to T cell receptors, the class II phosphatidylinositol 3 kinase C2β (PI3K-C2β) is activated, which, in turn, promotes production of PI(3)P (74). Several studies indicate that KCa3.1 channel activation by PI(3)P is associated with NDPK-B. The inhibitory effect of the 14 amino acid region at CT of KCa3.1 is eliminated upon recruitment of nucleoside diphosphate kinase B (NDPK-B) to phosphorylate the histidine residue H358 in this region (75). Based on the above mechanism, existing studies have focused on inhibition of KCa3.1 channel opening through potential effects on three sites of activity. First, intracellular PI (3)P synthesis is restricted by the PI(3)P phosphatase myotubularin-related protein 6 (MTMR6). Consistently, the highly selective PI3K inhibitor wortmannin depletes intracellular PI(3)P that results in inhibition of KCa3.1 (75, 76). Second, phosphoglycerate mutase family 5 (PGAM5) induces dephosphorylation of NDPK-B and directly inhibits NDPK-B-mediated histidine phosphorylation, thereby blocking KCa3.1 channel activation (77). Third, the mammalian protein histidine phosphatase (PHPT-1) binds directly to phosphorylated H358, triggering its dephosphorylation to achieve inhibition of KCa3.1 channel activity (78). Moreover, intracellular copper deficiency is associated with elevated H358 phosphorylation, implying that the use of copper chelators may enhance the activity of KCa3.1 (79). In an established KCa3.1^-/-^ mouse model, it was observed that T helper (Th)-0, Th1, and Th2 cells isolated from KCa3.1^-/-^ mouse are defective in Ca^2+^ flux and cytokine production, while the Th17 and Treg subsets displayed normal function. The above phenomenon supports a key role of KCa3.1 in Th0, Th1, and Th2-mediated diseases, including RA, colitis, and several other immune inflammatory disorders (80). Consistently, pharmacological inhibition of KCa3.1 decreased inflammatory bowel disease mice symptoms *via* increasing IL-10 production in T_reg_ cells, suggests that KCa3.1 is responsible for the invalidation of anti-inflammatory efficiency of T_reg_ cells in chronic inflammatory disorders (81, 82).

CD8^+^ cells are cytotoxic T lymphocytes that infiltrate solid tumors to perform immune surveillance functions. Chimote et al. provided evidence of compartmental reduction of CaM levels at the plasma membrane of CD8^+^ T cells in head and neck squamous cell carcinoma (HNSCC) patients, leading to decreased activity and chemotaxis of KCa3.1 (83). Similarly, another recent study showed that targeted KCa3.1 activation could restore the chemotaxis ability of HNSCC CD8^+^ T cells in the presence of adenosine (84). Furthermore, KCa3.1 is reported to support the migration of CD8^+^ T cells. Reduced K^+^ channel activity could be restored by cytokines, ultimately leading to functional recovery of impaired CD8^+^ T cells, facilitating clearance of pathogens or control of local tissue inflammation (85).

### Role of KCa3.1 in B cells

The primary function of B cells is to differentiate into plasma cells that secrete antibodies to mediate humoral immune response under conditions of antigen stimulation and Th cell assistance. In RA condition, abnormal activation of B cells lead to autoantibodies secretion following autoantigen presentation by certein antigen presenting cells. In addition, B cells can regulate bone formation in RA by inhibiting differentiation of osteoblasts (86). Other than supporting T cell proliferation, KCa3.1 coordinates the proliferation and migration of B cells. KCa3.1 is reported to be expressed in B cells and activity of the channel is significantly elevated during differentiation of activated naive B cells into memory B cells (87, 88). As professional antigen-presenting cells, B cells play a significant role in the adaptive immune response. Mechanistically, B cells ingest, process, and present antigens by expressing the B cell receptor (BCR) and regulating the human leukocyte antigen HLA-DO (89). Non-competitive anti-N-methyl-D-aspartate-receptor (NMDAR) antagonists modulate BCR-induced B cell proliferation, migration, and production of the anti-inflammatory factor interleukin-10 (IL-10) through negative regulation of the KCa3.1 channel (88). *KCNN4* encoding KCa3.1 has been characterized as a tissue-specific transcriptional coactivator (OCA-B)-dependent gene involved in B cell proliferation and function that is required for antigen-dependent B cell differentiation.

In contrast to the above findings, KCa3.1 has been shown to be positively engaged in BCR-induced B cell proliferation but not required during the active phase of B cell differentiation (90). After TRAM-34 treatment, the ability of B lymphocytes to proliferate was weaker and expression of chemokine (C-C motif) ligand 7, a chemotactic-related factor that promotes B cell migration, significantly decreased (91). While the underlying mechanisms have not been established, it is reasonable to speculate that Ca^2+^-associated changes are significantly linked to inhibition of KCa3.1 channel in B cells. At the molecular level, activation of the extracellular signal-regulated kinase (ERK) upstream protein RAS affects the ERK signaling pathway, leading to reduced secretion of B cell chemokines and recruitment of inflammatory cells (92).

### Role of KCa3.1 in macrophages

Macrophages play a fundamental role in the pathogenesis of RA disease, with significant infiltration at the inflamed synovium and cartilage junction, promoting inflammation by secreting cytokines and chemokines (93). Studies demonstrated that macrophages may contribute to RA synovial inflammation through activation of Notch signaling, leading to M1 pro-inflammatory phenotype, or *via* c-Jun N-terminal kinase (JNK) signaling channels activating nuclear factor κB and producing large amounts of tumor necrosis factor-α (TNF-α) (94). Earlier *in vitro* studies have demonstrated KCa3.1 expression in macrophages, with key roles in regulation of macrophage proliferation, migration, reactive oxygen species (ROS), and cytokine production (95, 96). In keeping with its role in T and B cells, KCa3.1 is reported to maintain Ca^2+^ influx and membrane hyperpolarization in macrophages (97). Upon blockage of the KCa3.1 channel in a study by Xu et al., the activity of signal transducer and activator of transcription 1 (STAT-1) protein was inhibited and phosphorylation levels reduced in macrophages (98). Moreover, the levels of pro-inflammatory cytokines and chemokines were significantly decreased in M1 macrophages whereas markers in M2 macrophages remained unchanged, suggesting that the KCa3.1 channel mainly regulates the function of M1 type macrophages and expression of pro-inflammatory genes. In chronic diseases, such as RA, multinucleation of macrophages is a critical step in the formation, differentiation and activation of osteoclasts, which lead to bone erosion and long-term inflammation (99, 100). In a microarray analysis of fused rat macrophages and human monocytes forming osteoclasts by Kang et al., the role of *KCNN4* as a potential modulator of multinucleation was validated (69). The main downstream effect of nuclear factor-κB (NF-κB) activation is upregulation of T cell dephosphorylation by nuclear translocation of nuclear factor cytoplasmic 1 (NFATc1), which stimulates Ca^2+^ signaling and activates Akt. Silencing or blockage of KCa3.1 suppressed NFATc1 expression and Akt activation, implying that *KCNN4* is also closely associated with cell death (69). Another study reported that TNF-α mediates the NF-κB pathway through increased autocrine secretion. NF-κB binds directly to the promoter region of *KCNN4* and enhances its activity to upregulate gene expression and promote cell proliferation (101). Furthermore, blockade of the KCa3.1 channel with TRAM-34 negatively regulates NF-κB and STAT3 signaling and impairs the ability of macrophages to differentiate into the pro-inflammatory M1 phenotype, in parallel with reduced levels of inflammatory factors, such as interleukin-1 (IL-1), interleukin-6 (IL-6), TNF-α and monocyte chemoattractant protein-1 (MCP-1) (102). The majority of studies indicate that the role of KCa3.1 in macrophages is closely associated with NF-κB and STAT signaling pathways.

### Role of KCa3.1 in mast cells

Mast cells (MCs) recognize endogenous and exogenous mediators, which boost the release of various mediators from other immune and non-immune cells, consequently regulating different physiological activities *in vivo* (103). During the process of RA, activated MCs produce an array of pro-inflammatory mediators that activate other immune system cells, initiating and maintaining the inflammatory response. TNF-α preformed by mast cells initiates an inflammatory cascade response promoting cytokine expression. Meanwhile, products of mast cells, in particular, histamine and TNF-α, promote proliferation and catabolic effects of articular chondrocytes and synovial stromal cells, leading to the development of RA (104). A number of previous studies have confirmed the presence of KCa3.1 in mast cells. Activation of the KCa3.1 channel maintains high concentrations of intracellular free Ca^2+^ in mast cells, promotes IgE-dependent histamine release, and regulates the secretory responses of mast cells (105). The Orai/CRACM1 ion channel provides the major Ca^2+^ influx pathway for mast cells to release mediators and activation of the KCa3.1 channel in mast cells is highly dependent on this process (106). Prostaglandin E2 (PGE2) suppresses the IgE-dependent cell activation pathway by inhibiting activation of EP2 receptors. Inactivation of EP2 receptors limits the influx of free cytoplasmic Ca^2+^, leading to reduced chemokine production and subsequent closure of the KCa3.1 channel (107). Upon interference with channel gene expression *via* lentiviral targeting of KCa3.1, signaling pathways are disrupted and mast cell activity is reduced, followed by attenuation of the immune inflammatory response (108). In addition, E3 ubiquitin ligase (containing a tripartite motif of protein 27) negatively regulates high-affinity receptor for IgE (FcepsilonRI) activation and downstream signaling of KCa3.1 through ubiquitination and inhibition of PI3KC2β in mast cells (109). The levels of chemokine CXC motif chemokine ligand 10, chemokine stem cell factor, and TNF-α in mast cells are reported to be significantly decreased by charybdotoxin and TRAM-34, along with diminished mast cell migration capacity (110).

### Role of KCa3.1 in dendritic cells

Dendritic cells (DCs) participate in the presentation of autoantigens and production of pro-inflammatory factors, which contribute to ongoing inflammation in RA. In addition, DCs are in charge of maintenance and differentiation of autoimmune B and T cells which directly participated in RA pathogenesis (111). Studies show that the binding of lymphatic chemokines CCL19 and CCL21 to their receptor CCR7 induces mobilization of Ca^2+^ stored in mature DCs and subsequent opening of the KCa3.1 channel (112, 113). The migratory capacity of DCs is tightly regulated by the intracellular Ca^2+^ concentration and chemokine receptors are differentially expressed in DCs at two states of maturation. In the presence of TRAM-34, temporal coupling between KCa3.1 and Ca^2+^ inward flow was shown to be disrupted and subsequent CCR7-induced chemotaxis impaired (112). Paradoxically, KCa3.1 exhibited migratory capacity only in immature dendritic cells and expression of its migration marker CCR5 was modified in the presence of TRAM-34 (114, 115). Data from the above study additionally confirmed that activation of T lymphocyte proliferation by dendritic cells is not affected by KCa3.1. *In vitro*, prevention of [Ca^2+^]i elevation under conditions of KCa3.1 deficiency decreased the directed migration of lipopolysaccharide (LPS)-challenged DCs, supporting the involvement of KCa3.1 in LPS-induced DC migration (116).

### Roles of KCa3.1 in other immune cells

In RA, neutrophils can activate other immune cells that perpetuate inflammation and lead to the destruction of cartilage and bone in affected joints. This pathogenic effect occurs primarily through mechanisms including increased cell survival and migration capacity, abnormal inflammatory activity, elevated oxidative stress, and exacerbated neutrophil extracellular trap formation (117). Recently, Henríquez et al. demonstrated the existence of KCa3.1 in mammalian neutrophils for the first time and showed a positive correlation between upregulation of the channel and neutrophil migration (118). Concomitantly, targeted KCa3.1 inhibition altered the capacity of cells to properly regulate cell volume and limited neutrophil migration *in vitro* with no effect on Ca^2+^ homeostasis. Likewise, the membrane potential of the *KCNN4* ^-/-^ neutrophil subpopulation was balanced in a study by Grimes et al., resulting in a homogeneous lower-calcium (Ca^lo^) response (119). In addition, erythrocytes have a partial immune function although they are not conventional immune cells. The KCa3.1 channel present on erythrocytes regulates cellular volume by transporting K^+^ across the membrane and its activity increases in response to high cytokine levels (120). The role of KCa3.1 in immune cells has been summarized in the **Table 2**.

**Table 2:** Role of KCa3.1 in immune cell.

Cells	Experimental cells	Inhibition/activation of KCa3.1	Mechanism	References
**T lymphocytes**	CD4^+^ T cells	MTMR6/ Wortmannin	PI(3)P↓, KCa3.1↓, proliferation↓	(75, 76)
	CD4^+^ T cells	PGAM5	Dephosphorylation NDPK-B, Histidine phosphorylation↓, KCa3.1↑	(77)
	CD4^+^ T cells	PHPT-1	Bind p-H358, dephosphorylation p-H358, KCa3.1↓	(78)
	CD8^+^ T cells	1-EBIO	Chemotactic capacity↑	(84)
**B lymphocytes**	Splenic B cells	NMDAR antagonists	Inhibit BCR, KCa3.1↓, IgM, IgG↓, IL-10↑	(88)
	Splenic B cells	TRAM-34	KCa3.1↓, cells proliferation↓, CCL7↓, migration↓	(91)
**Macrophages**	THP-1 cells	TRAM-34	KCa3.1↓, STAT-1↓, type M1 polarization↓	(98)
	Human macrophages	*KCNN4* deficiency	KCa3.1↓, RANKL↓, NF-κB↓, NFATc1↓, Akt↑	(69)
**Mast cells**	HLMC	AH6809	EP2↓, KCa3.1↓, chemokine↓, migration↓	(107)
	P815 cells	LV-KCa3.1-shRNA	KCa3.1↓, AKT phosphorylation↓, IL-6, IL-8↓, mast cell activity↓	(108)
	BMMC	TRIM27^-/-^	FcϵR1↑, PI3KC2β↑, KCa3.1↑, mast cell activation↑	(121)
	HLMC	TRAM-34	KCa3.1↓, CXCL10, TNF-α↓, migration↓	(110)
**Dendritic cells**	Lung dendritic cells	TRAM-34	CCR7 inhibition, KCa3.1↓, migration↓	(112)
	Immature dendritic cells	TRAM-34	CCR5 inhibition, KCa3.1↓, migration↓	(114)

The symbols ↑ and ↓ mean the up-regulation and down-regulation of KCa3.1 expression, respectively.

## Correlative regulation of KCa3.1 and immune-inflammatory cytokines

**Figure 2:**
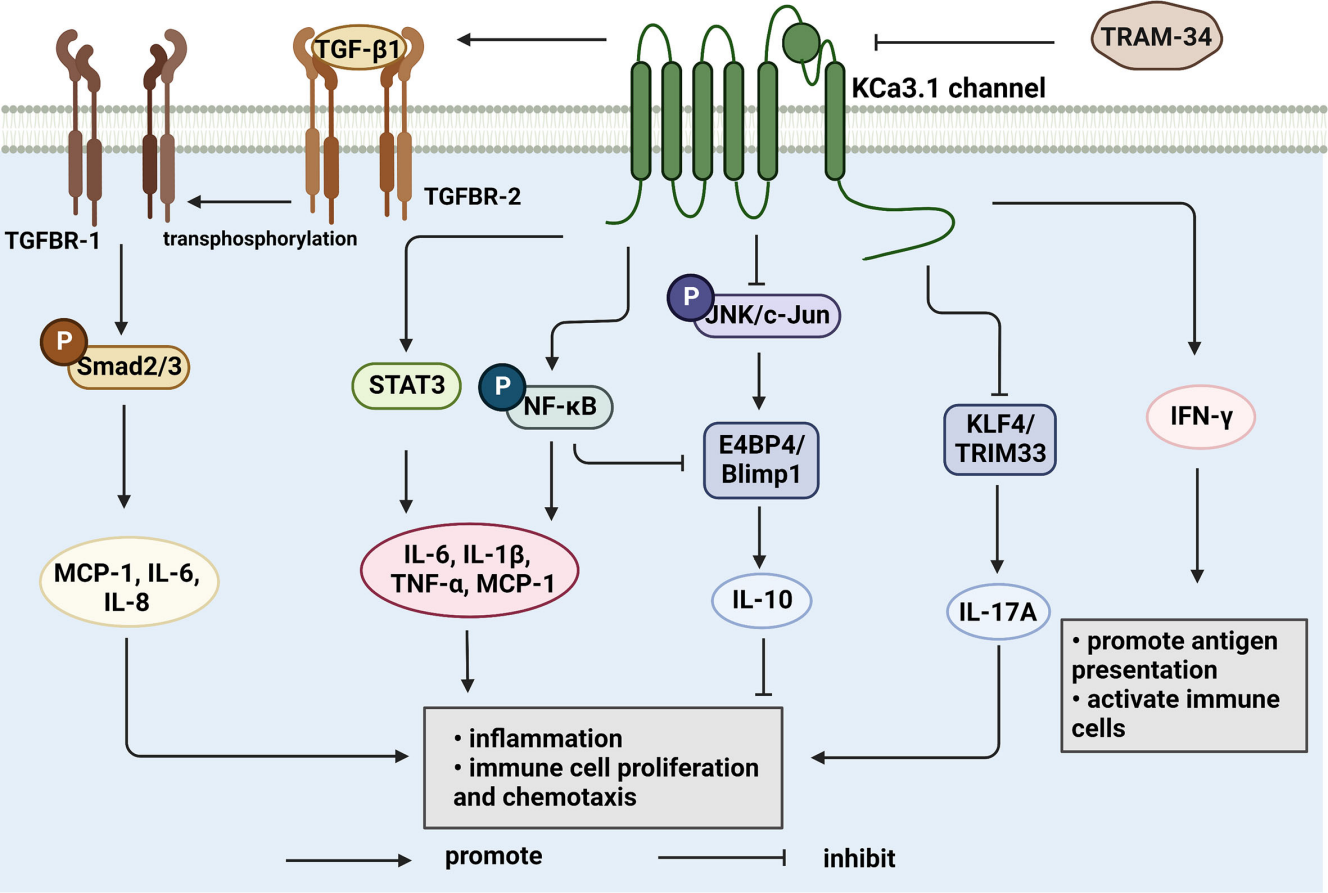
KCa3.1 regulates cytokine production and secretion. TGF-β1 binds to type II receptors and transphosphorylates type I receptors, phosphorylates Smad2/3 and secretes many inflammatory factors. Activation of KCa3.1 also promotes the secretion of IL-1β, IL-6, IL-8, TNF-α, and MCP-1 through the STAT3 and NF-κB signaling pathways. IFN-γ is upregulated by KCa3.1 either. KCa3.1 restrains the production of IL-10 through the JNK/c-Jun and NF-κB pathways. The blocked KCa3.1 channel by TRAM-34 inhibits these pathways. IL-17A is a pro-inflammatory cytokine that is upregulated by TRAM-34 through activation of KLF4 and/or TRIM33. (Created with BioRender.com).

Synovial inflammation is a critical process in the pathogenesis of RA and directly associated with clinical symptoms, such as inflammatory pain, joint swelling and progressive destruction of multiple joints. Accumulating evidence suggests that the KCa3.1 channel is capable of cytokine regulation with potential significant implications in immune-inflammatory diseases (**Figure 2**). KCa3.1 has been shown to stimulate TGF-β1 production. In experiments by C. Huang et al., treatment with TRAM-34 suppressed transcription of TGF-β1 and TGF-β1 type II receptor mRNA and negatively regulated phosphorylation of Smad2/3 (122). The above processes led to reduced production of inflammatory cytokines, PAI-1, and matrix proteins in the nucleus, with anti-inflammatory and anti-fibrotic effects. The KCa3.1 channel is reported to mediate K^+^ efflux, promote intracellular Ca^2+^ concentrations, and activate calmodulin kinase IV (CaMKIV), which facilitates CREB phosphorylation, contributing to upregulation of c-fos/AP-1 and NFATc1 expression, and ultimately leading to osteoclast formation (123). Moreover, NF-κB and STAT3 signaling pathways are inactivated upon blockade of the KCa3.1 channel. Consequently, decreased secretion of pro-inflammatory factors, such as IL-1β, IL-6, TNF-α, and MCP-1, limits the progression of inflammation (102). In regulatory T cells, suppression of KCa3.1 channel activity initiates phosphorylation of JNK and c-Jun, activation of JNK/c-Jun signaling, and E4BP4/Blimp1-mediated anti-inflammatory IL-10 cytokine secretion (81, 82). The above findings suggest that inhibition of KCa3.1 channel activity modulates immune-inflammatory factors and alleviates inflammation. Paradoxically, TRAM-34 is reported to activate two types of transcriptional regulators, KLF4 and/or TRIM33, and mediate upregulation of pro-inflammatory IL-17A (82). Another study disclosed no pro-inflammatory changes in T cell subsets and plasma cytokines or chemokines following administration of SKA-31, a KCa3.1 activator, in rats (124).

**Figure 3:**
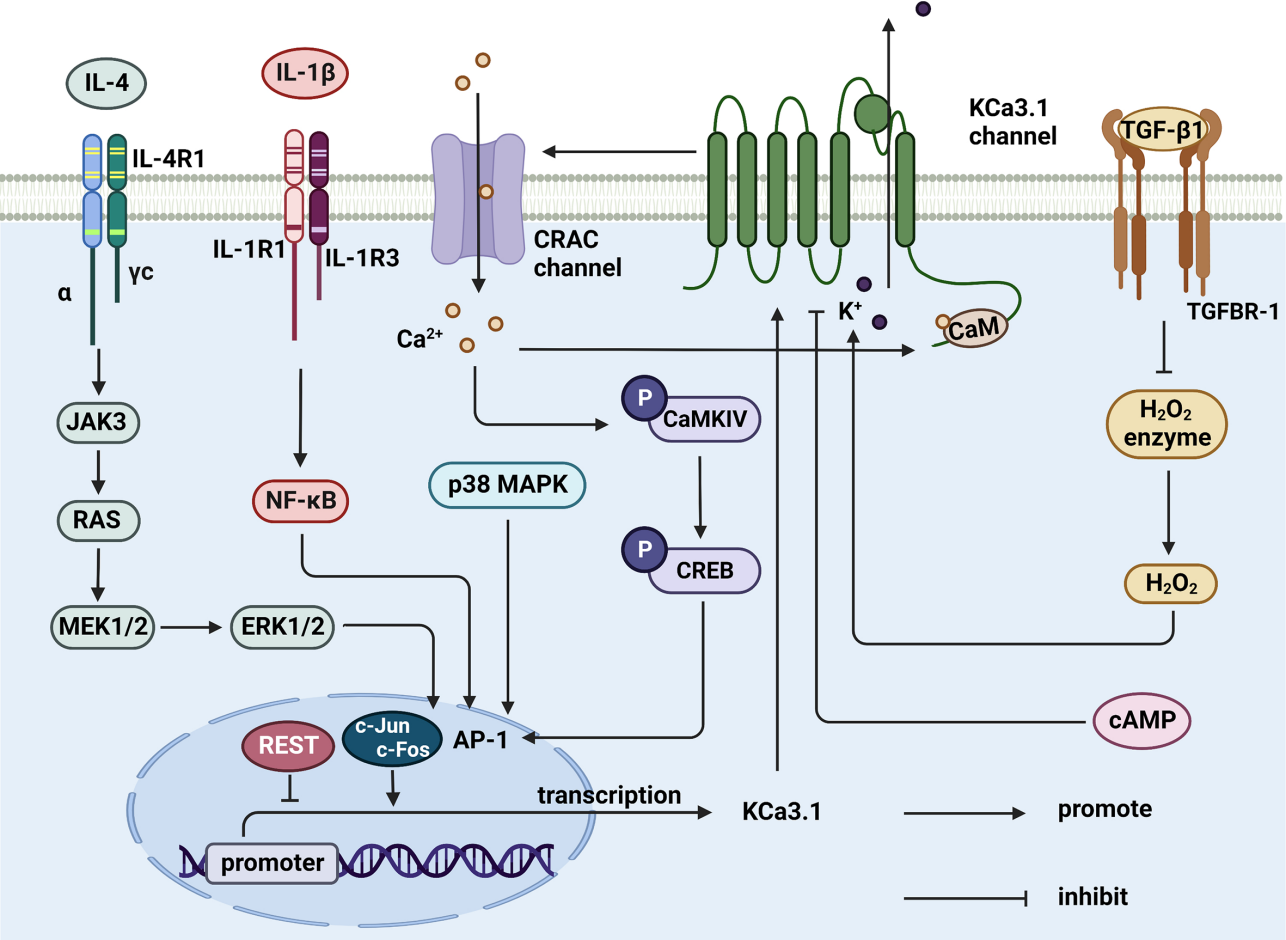
Cytokines regulate KCa3.1 expression and activity. TGF-β1 inhibits catalase, thereby synthesizing hydrogen peroxide to activate KCa3.1. IL-4 and IL-1β upregulates AP-1 through JAK3/RAS/MEK/ERK and NF-κB signaling pathway, resulting in the initiation of KCa3.1 transcription. AP-1 can be upregulated by activated CaMKIV/CREB and p38 MAPK pathway, either. KCa3.1 activity is controlled by elevated cAMP in response to agents. (Created with BioRender.com).

The KCa3.1 channel both regulates and is regulated by cytokines (**Figure 3**). TGF-β1 has the capability to inhibit catalase activity and promote hydrogen peroxide levels, thereby inducing an increase in KCa3.1 expression. The p38MAPK signaling pathway plays a vital role in stress responses, such as inflammation and apoptosis. p38MAPK/AP-1/NF-κB signaling activates the AP1 complex (composed of c-fos and c-Jun) and promotes transcription and translation of KCa3.1 (125). Upregulation of KCa3.1 stimulates the expression and production of interferon-γ (IFN-γ), in turn, mediating the mobilization and accumulation of inflammatory T cells, which are involved in inflammation (126). In addition, IL-1β stimulation is reported to activate NF-κB signaling and upregulate the KCa3.1 channel in pancreatic islet cells. The drug modafinil suppresses progression of inflammation *via* elevation of adenosine 3’, 5 cyclic monophosphate (cAMP) and inhibition of KCa3.1 channel activity (127). Furthermore, IL-4 specifically binds type I receptors and regulates JAK3 and RAS/MEK/ERK signaling pathways. In the above mechanisms, the transcription factor AP-1 is activated and upregulates KCa3.1 (128). However, in-depth studies revealed that IL-4 increases the current in the KCa3.1 channel only slightly, inducing no significant changes in channel density with increasing membrane area (40). Based on the available information, targeting the KCa3.1 channel is proposed as a means to effectively regulate immune-related molecules, such as cytokines and inflammatory factors, which play a crucial part in immune system-mediated disorders.

## KCa3.1 as a potential drug target for RA

### Targeting the inflammatory process of RA

The occurrence and continuous development of RA is manifested by failure of spontaneous regression of inflammation. Increasing evidence suggests that KCa3.1 promotes secretion of inflammatory factors by regulating immune-inflammatory cells in RA. In related reports, KCa3.1 is considered a pro-inflammatory ion channel that activates the function of inflammasome. Hydroxychloroquine is reported to impair the inflammasome and inhibit neutrophil recruitment in a dose-dependent manner through inhibition of Ca^2+^-activated K^+^ conductance in THP-1 macrophages (70). Interestingly, earlier findings indicate that TGF-β induces transcription and translation of KCa3.1 and, conversely, silencing or inhibition of KCa3.1 negatively regulates TGF-β (8). In addition, the pro-inflammatory and invasive behavior of synovial fibroblasts plays an essential role in RA. Another study showed that blockage of KCa3.1 with TRAM-34 or siRNA treatment could suppress proliferation of RA-SFs. Inactivation of the channel led to downregulation of the pro-inflammatory factors IL-6, interleukin-8 (IL-8), and MCP-1, as well as tissue-destructive protease MMP3 at both mRNA and protein levels. Notably, inhibition of the KCa3.1 channel also upregulated MMP1 mRNA and enhanced secretion of IL-1β while decreasing that of IL1-RA, resulting in inhibition of short-term activation of Th2 lymphocytes in RA and consequently, a shift in the inflammatory homeostasis of RA to a pro-inflammatory state (41). However, limited data on the specific role of KCa3.1 in inflammation of RA are available at present. Further studies are required to elucidate the functions and mechanisms of action of KCa3.1 in the inflammatory process associated with RA.

### Targeting of cartilage destruction and bone erosion

The pathogenesis of RA is synovial inflammation accompanied by cartilage damage and bone erosion. In addition to synovial tissue and immune cells that show critical immune-inflammatory activities, synovial fibroblasts and osteoclasts play a central role in cartilage and bone destruction and bone erosion in RA. Although no evidence of direct mediation of RA cartilage and osteogenic destruction by KCa3.1 has been obtained, its involvement in these processes *via* regulation of fibroblast (FLS) and osteoclast activation is a strong possibility.

Previous studies have shown that highly activated FLS can promote inflammation and tissue invasion and mediate tissue damage with tissue-infiltrating macrophages and immune cells, such as T cells and B cells (42). FLS are involved in the pathological process of synovitis, synovial lining hyperplasia, activation of a number of synovial cells, and destruction of cartilage matrix through production of cytokines and chemokines. The p38 MAPK (mitogen-activated protein kinase) pathway is a crucial signal transduction step during chronic inflammation (49). Two isoforms of p38MAPK, α and γ, are expressed in FLS, which play key roles in the inflammatory process by activating the p38MAPK signaling pathway to produce inflammatory factors, such as TNF-α, IL-1β and IL-6 (129). In addition, FLS regulate the proliferation and differentiation of immune cells through the p38 pathway. Transcriptional growth factor β1 (TNF-β1) is highly expressed in RA-SFs and can induce expression of pro-inflammatory and pro-destructive proteins (130). TNF-β1 has been shown to induce *KCNN4* transcription and translation, activate the KCa3.1 channel, increase K^+^ current, provide continuous power for Ca^2+^ influx, and promote inflammatory processes (131). At present, studies on the mechanism of action of KCa3.1 in synovial fibroblasts are lacking and the pathways underlying KCa3.1 upregulation by TGF-β1 remain to be established. Further clarification of whether KCa3.1 has functional activity in RA-SFs through signaling pathways, such as p38MAPK (132) and NF-κB (133, 134), should further support its potential involvement in RA cartilage injury.

Osteoclasts and RANKL act together to promote the occurrence of bone erosion (124, 125). In a recent study, activation of endogenous fibroblast-like synoviocytes induced RANKL expression and stimulated osteoclast formation (135, 136). The KCa3.1 channel inhibitors, TRAM-34 and ICA-17043, have been shown to inhibit monocyte formation in osteoclasts in a dose-dependent manner but the precise molecular mechanisms remain to be established (69). It is speculated that the KCa3.1 channel is functionally active in the formation of osteoclasts. KCa3.1 can prevent the progression of bone erosion by inhibiting the differentiation and formation of osteoclasts, thereby relieving the clinical symptoms of RA patients, providing further support for its utility in management of RA.

## Blockage of the KCa3.1 channel

In applications of KCa3.1 channel inhibitors, existing studies indicate that TRAM-34 exerts no notable side-effects when used at a high concentration (~120 mg/kg) and has no effect on blood biochemistry and hematology parameters (137). Senicapoc has passed Phase I-III clinical trials for clinical drug use in sickle cell disease, with a reported IC_50_ value of 11 nm (138). Senicapoc may cause diarrhea, nausea, and other adverse reactions in a dose-dependent manner, but overall drug safety is good. The KCa3.1 inhibitors TRAM-34 and Senicapoc have been used in RA-related *in vitro* studies (8, 69). Clotrimazole and nitrendipine have progressed to the clinical trial stage and are widely used to treat a number of diseases. According to the tissue distribution characteristics of the KCa3.1 channel, KCa3.1 generally not expressed in excitable tissues and reproductive organs, which indicates a low-risk, acute-toxicology profile of KCa3.1 channel blockade. The results obtained to date support the feasibility, efficacy, and safety of the KCa3.1 channel as a therapeutic target in RA. However, extensive research is required before introduction of KCa3.1 channel blockers in the clinic. Remarkably, related studies have shown that the KCa3.1 channel is the basis of slow afterhyperpolarization (SAHP) in neurons and may exert side-effects that affect sensory transmission (139).

## Conclusions and outlook

**Figure 4:**
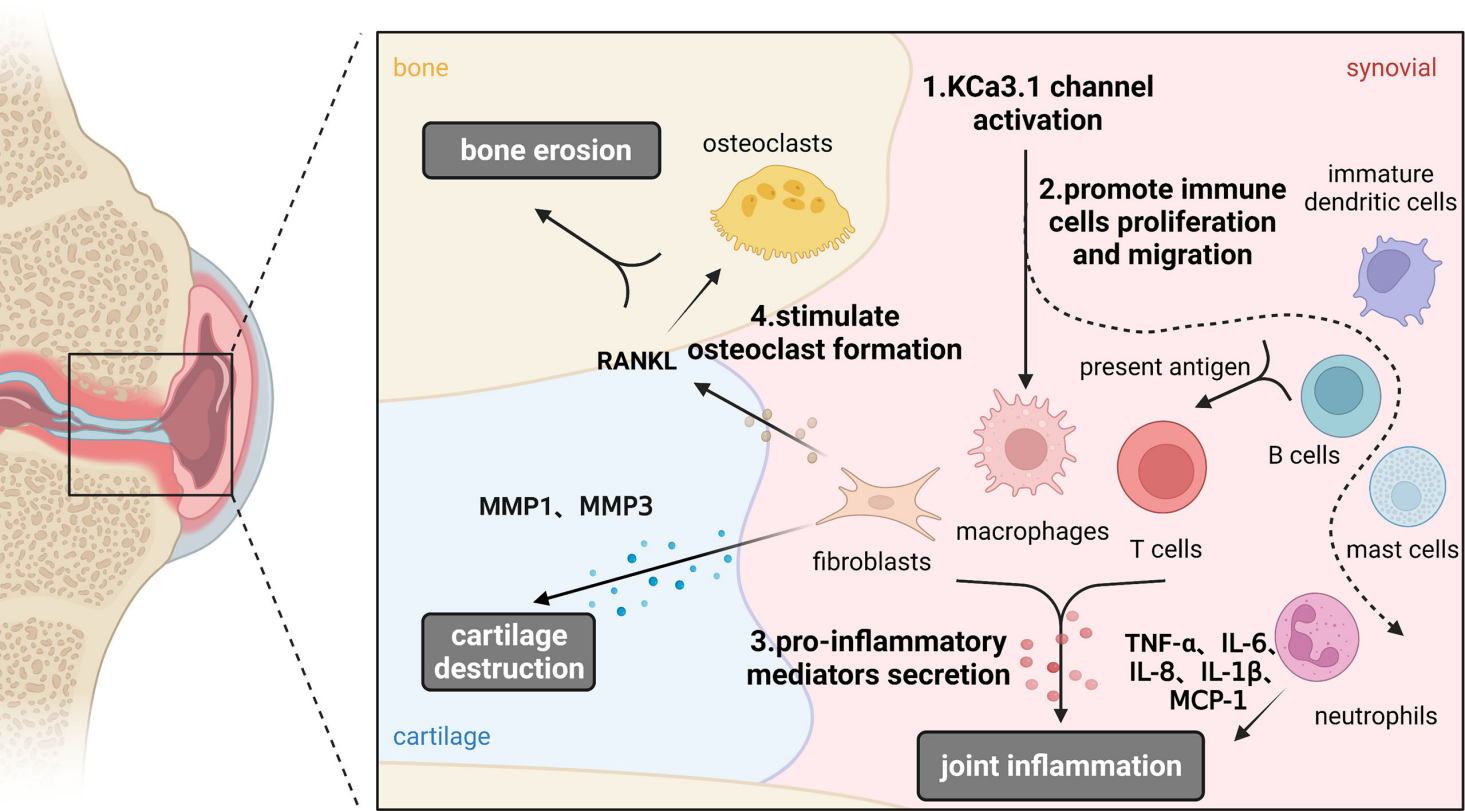
Functions of KCa3.1 channel in the pathological process of rheumatoid arthritis. KCa3.1 is involved in RA inflammation, cartilage and bone destruction by regulating the abnormal activation of immune cells, synoviocytes and osteoclasts. Dashed arrows indicate possible mechanisms in rheumatoid arthritis. (Created with BioRender.com).

KCa3.1 promotes inflammation, cartilage damage, and bone erosion in synovial fibroblasts and osteoclasts that are mechanistically involved in development of RA. Based on its ability to restore the immune balance by interfering with Ca^2+^ signaling, KCa3.1 presents a promising therapeutic target for RA. The possible functions of KCa3.1 in the pathological process of RA is shown in **Figure 4**. Despite interesting experimental findings to date, research in this field is still in its infancy. Considerable work remains to be done to elucidate the in-depth mechanisms underlying the involvement of KCa3.1 in RA. For example, the issue of whether KCa3.1 directly mediates cartilage and bone destruction in RA is yet to be resolved. Furthermore, no clinical trials have directly investigated the effects of KCa3.1-specific inhibitors and activators in RA as yet. In summary, KCa3.1 provides excellent research prospects for treatment of RA and further development of drugs targeting this channel may be of considerable benefit to patients.

## Author contributions

RP-Z and WH conceived this project. LY and Y-JZ prepared the first draft. H-LZ, W-JH, R-DZ, YW and R-PZ revised the manuscript. All authors contributed to the article and approved the submitted version.

## Funding

This work was supported by grants from the National Natural Science Foundation of China (82272450, 81902182, 82071591) and the Natural Science Foundation Incubation Program of The Second Hospital of Anhui Medical University (2021GMFY06).

## Conflict of interest

The authors declare that the research was conducted in the absence of any commercial or financial relationships that could be construed as a potential conflict of interest.

## Publisher’s note

All claims expressed in this article are solely those of the authors and do not necessarily represent those of their affiliated organizations, or those of the publisher, the editors and the reviewers. Any product that may be evaluated in this article, or claim that may be made by its manufacturer, is not guaranteed or endorsed by the publisher.

## Glossary

**Table:**

1-EBIO	1-Ethyl-2-benzimidazolidinone
AP-1	activation protein-1
BCR	B cell receptor
BMMC	bone marrow-derived mast cells
CAIA	collagen antibody-induced arthritis
CaM	calmodulin
CAMBD	CAM-binding domain
CaMKIV	calmodulin kinase IV
cAMP	adenosine 3’, 5 cyclic monophosphate
CHO cells	Chinese hamster ovary cells
CIA	collagen-induced arthritis
CRE	cAMP response element
CVID	common variable immunodeficiency
CZ	chlorzoxazone
DCs	dendritic cells
DC-EBIO	5, 6-dichloro-1-EBIO
DMARDs	disease-modifying anti-rheumatic drugs
EC_50_	effective concentration producing 50% of maximum response
FLS	fibroblast-like synoviocytes
GC	glucocorticoids
H-89	N-[2-(4-bromocinnamylamino) ethyl]-5-isoquinoline
HDAC	histone deacetylase
HEK-293 cells	human embryonic kidney cells
HNSCC	head and neck squamous cell carcinoma
IBMX	3-isobutyl-1-methylxanthine
IC_50_	inhibitory concentration (e.g. 50% inhibition of maximum)
IFN-γ	interferon-γ
IGFBP5	insulin-like growth factor-binding protein 5
IL	interleukin
JNK	c-Jun N-terminal kinase
KCa3.1	intermediate conductance Ca^2+^-activated K^+^ channel
LPS	lipopolysaccharide
LZ	leucine zipper
MAPK	mitogen-activated protein kinase
MCP-1	monocyte chemoattractant protein-1
MCs	mast cells
MGCs	multinucleated macrophages
MTMR6	myotubularin-related protein 6
MTX	Maurotoxin
NDPK-B	nucleoside diphosphate kinase B
NFATc1	nuclear translocation of nuclear factor cytoplasmic 1
NF-κB	nuclear factor-κB
NMDAR	anti-N-methyl-D-aspartate-receptor
NS309	6, 7-dichloro-1H-indole-2, 3-dione-3-oxime
NS6180	4-[[3-(trifluoromethyl) phenyl] methyl]-2H-1, 4-benzothiazin-3(4H)-one
NSAIDs	non-steroidal anti-inflammatory drugs
PGAM5	phosphoglycerate mutase family 5
PGE2	prostaglandin E2
PHPT-1	protein histidine phosphatase
PI3K	phosphatidylinositol 3-kinase
PI3K-C2β	the class II phosphatidylinositol 3 kinase C2β
PI(3)P	phosphatidylinositol 3 phosphate
PKA	cAMP-dependent protein kinase
RA	rheumatoid arthritis
RANKL	receptor activator of nuclear factor-κB ligand
REST	repressor element 1-silencing transcription
SKA-121	5-methylnaphtho [2, 1-d] oxazol-2-amine
SKA-31	Naphtho [1, 2-d] thiazole-2-ylamine
STAT-1	signal transducer and activator of transcription 1
TNF-α	tumor necrosis factor-α
TNF-β1	transcriptional growth factor β1
TRAM-34	1-[(2-chlorophenyl) diphenylmethyl]-1H-pyrazole
TRIM27	tripartite motif containing protein 27
ZOX	zoxazolamide
